# Supplementary material for: Complex‐centric proteome profiling by SEC‐SWATH‐MS
Source: Mol Syst Biol. 2019 Jan 14;15(1):e8438. doi: 10.15252/msb.20188438 (PMC6346213; doi:10.15252/msb.20188438)
Supplement: Supplementary file 8 — Dataset EV7 [file MSB-15-e8438-s008.zip › feature_plots_string/Q9UEU0.pdf]

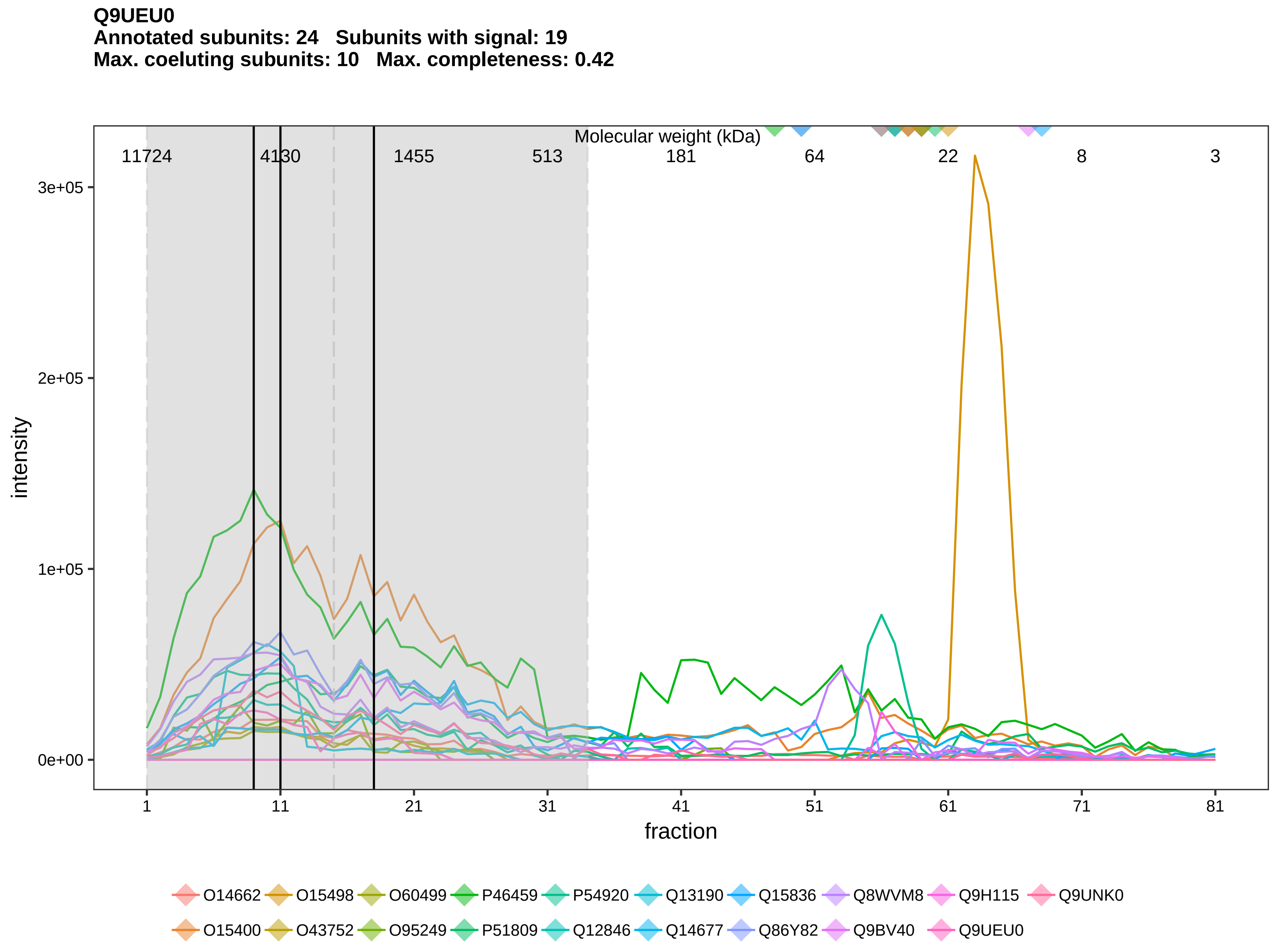

Q9UEU0
Annotated subunits: 24 Subunits with signal: 19
Max. coeluting subunits: 10 Max. completeness: 0.42
Molecular weight (kDa)
11724
4130
1455
513
181
64
22
8
3
3e+05
2e+05
1e+05
0e+00
intensity
1
11
21
31
41
51
61
71
81
fraction
O14662
O15498
O60499
P46459
P54920
Q13190
Q15836
Q8WVM8
Q9H115
Q9UNK0
O15400
O43752
O95249
P51809
Q12846
Q14677
Q86Y82
Q9BV40
Q9UEU0
